# Adjusting microwave sensing frequency through aspect ratio variation and bending repetitions in Permalloy ellipses

**DOI:** 10.1038/s41598-024-66802-7

**Published:** 2024-07-24

**Authors:** Nayeon Kim, Dongpyo Seo, ByungRo Kim, Youjung Kim, Seungha Yoon, Jin Hyeok Kim

**Affiliations:** 1 Optoelectronics Convergence Research Center and Department of Materials Science and Engineering, Chonnam National University, Gwangju, 61186 Republic of Korea; 2 School of Materials Science and Engineering, Gwangju Institute of Science and Technology, Gwangju, 61005 Republic of Korea; 3 Energy & Nano Technology R&D Group, Korea Institute of Industrial Technology, Gwangju, 61012 Republic of Korea

**Keywords:** Ferromagnetic resonance, Permalloy ellipse, Aspect ratio, Flexible, Applied physics, Sensors and biosensors

## Abstract

The Ferromagnetic Resonance (FMR) phenomenon, marked by the selective absorption of microwave radiation by magnetic materials in the presence of a magnetic field, plays a pivotal role in the development of radar absorbing materials, high speed magnetic storage, and magnetic sensors. This process is integral for technologies requiring precise control over microwave absorption frequencies. We explored how variations in resonance fields can be effectively modulated by adjusting both the shape and stress anisotropies of magnetic materials on a flexible substrate. Utilizing polyethylene-naphthalate (PEN) as the substrate and Permalloy (Ni_79_Fe_21_, noted for its positive magnetostriction coefficient) as the magnetic component, we demonstrated that modifications in the aspect ratio and bending repetitions can significantly alter the resonance field. The results, consistent with Kittel’s equation and the predictions of a uniaxial magnetic anisotropy model, underscore the potential for flexible substrates in enhancing the sensitivity and versatility of RF-based magnetic devices.

## Introduction

Ferromagnetic resonance (FMR)^1–5^ not only stands as a pivotal measurement technique for probing into the fundamental magnetic properties but also emerges as a significant contender for applications across radar absorbing materials^6–8^, ultra-fast magnetic storage, and sensing devices^9–11^. One remarkable application is seen in the development of stealth technology for military aircraft. Incorporating FMR materials into the outer coatings of aircraft allows for the effective absorption of radar signals, rendering the aircraft less detectable. This application is underpinned by the ability of FMR absorbers to selectively absorb specific frequencies, making them ideal for countering radar systems operating within those bands. Furthermore, the field of ultrafast magnetic storage devices benefits significantly from advancements in FMR technology. The precision tuning of magnetic properties, facilitated by an in depth understanding of magnetic anisotropy and FMR dynamics, enables the development of storage media with faster read/write cycles and higher data density. Sensing devices, particularly those used in environmental monitoring and biomedical applications, have seen remarkable improvements through the integration of FMR techniques. For example, magnetic sensors equipped with FMR capabilities can detect minute changes in magnetic fields, allowing for the sensitive detection of pollutants or biomarkers at very low concentrations.

FMR is characterized by the enhanced absorption of microwave energy when exposed to an effective magnetic field alongside electromagnetic waves, coinciding with the natural precession frequency of magnetic moments within materials. The utilization of FMR based microwave absorbers is primarily in the diminution and absorption of unwanted microwave signals across diverse applications^12–16^, showcasing their adaptability and critical role in modern technology. These absorbers are meticulously crafted using materials specifically chosen for their frequency characteristics, enabling selective absorption of microwaves. This selective behavior allows them to reflect or transmit other frequencies, thereby acting as a pivotal component in a variety of electronic and communication devices. Moreover, the adjustability of the absorption frequency is made possible by incorporating various forms of magnetic anisotropy, including crystalline, shape, and stress anisotropy, into the thin films and patterns^17–22^. This adaptability underscores the potential for FMR technology in creating highly efficient and customizable microwave absorbers.

The theoretical underpinning for understanding these variations in the absorption frequency is encapsulated in Kittel’s equation^23–27^. This equation lays the groundwork for predicting how alterations in magnetic anisotropy can systematically influence the ferromagnetic resonance frequency. The equation thus serves as a critical tool in the design and optimization of FMR-based devices, guiding the development of materials and structures that can be finely tuned for specific applications. By leveraging this theoretical framework, researchers can engineer materials with predetermined magnetic properties, opening new avenues for advancements in radar technology, data storage solutions, and sensor development. The theoretical framework for understanding variations in absorption frequency $\left( {f_{res} } \right)$ is provided by Kittel’s equation, as follows:

1 $$f_{res} = \gamma_{e} \sqrt {\left( {H_{0} + H_{eff} } \right)\left( {H_{0} + 4\pi M_{S} } \right)}$$

Here, *γ*_*e*_ represents the electron’s gyromagnetic ratio of 28.024952 [GHz/T]. H_0_ is the external magnetic field, and *H*_*eff*_ is the sum of the magnetic anisotropy fields, including contributions from crystalline, shape, and magnetostriction effects. Adjusting the dimensions of magnetic elements to influence shape anisotropy plays a pivotal role in meticulously fine tuning coercivity, bolstering magnetic stability, and steering the magnetization dynamics. These adjustments are indispensable in the engineering and design of radio frequency (RF) absorbing devices, as they have a profound impact on the performance of these devices^28–31^. Moreover, the magneto-mechanical effect, which is closely related to stress anisotropy, stands as a cornerstone for deciphering how resonance frequency is influenced by the direction of applied stress and the frequency of bending repetitions in flexible magnetic thin films and patterns^32–39^.

In this study, we delved into how the absorption frequency can be finely manipulated by concurrently adjusting both shape and stress anisotropy in arrays of Permalloy ellipses. Permalloy (Ni_79_Fe_21_), a material celebrated for its negligible magneto-mechanical response, showcases a magnetostriction coefficient of approximately ~ $10^{ - 6}$^40^. By modulating the aspect ratio, we successfully altered the FMR response in a systematic manner. This method not only illustrates Permalloy’s sensitivity to subtle variations in physical dimensions and applied stresses but also highlights the immense potential for precision tuning of magnetic resonance properties in the burgeoning field of flexible spintronic devices. Our investigation demonstrates the relationship between physical shape and mechanical stress on permalloy, highlighting its potential for developing future RF absorbing devices. The ability to fine tune the magnetic resonance through such manipulations opens up new possibilities for creating more efficient, adaptable, and high performing flexible electronic devices. This study paves the way for leveraging the unique properties of Permalloy and similar materials in the design of next generation devices that require precise control over their magnetic behavior for applications in various fields including, but not limited to, flexible electronics, wearable technology, and advanced communication systems.

## Experimental

Ellipse arrays were meticulously crafted employing conventional photolithography technique before being deposited onto 12 mm × 12 mm polyethylene-naphthalate (PEN) substrates using dc magnetron sputtering. The photolithography process involved coating the substrates with AZ GXR 601 photoresist at a spin speed of 4000 rpm, followed by baking at 180 °C for 90 s to enhance the photoresist’s attachment to the PEN substrates and to ready them for UV light exposure. The exposure to UV light lasted for 7 s, after which the ellipse array patterns were developed using MIF 300 for 30 s and subsequently rinsed with deionized (DI) water for 90 s to ensure cleanliness. During the sputtering process, an external magnetic field of about 20 mT was applied to the PEN substrate along the y-axis, courtesy of the permanent magnets embedded in the sample holder. This meticulous setup ensured the orientation of the deposited magnetic materials. The fabricated layers consisted of 27 nm of Permalloy and a protective as well as adhesive 3.6 nm layer of tantalum at both the bottom and top. The inclusion of tantalum layers significantly enhances the structural integrity of the ellipse arrays by improving adhesion to the substrate and providing crucial protection against oxidation, thereby ensuring the longevity and reliability of the magnetic properties.

**Figure 1:**
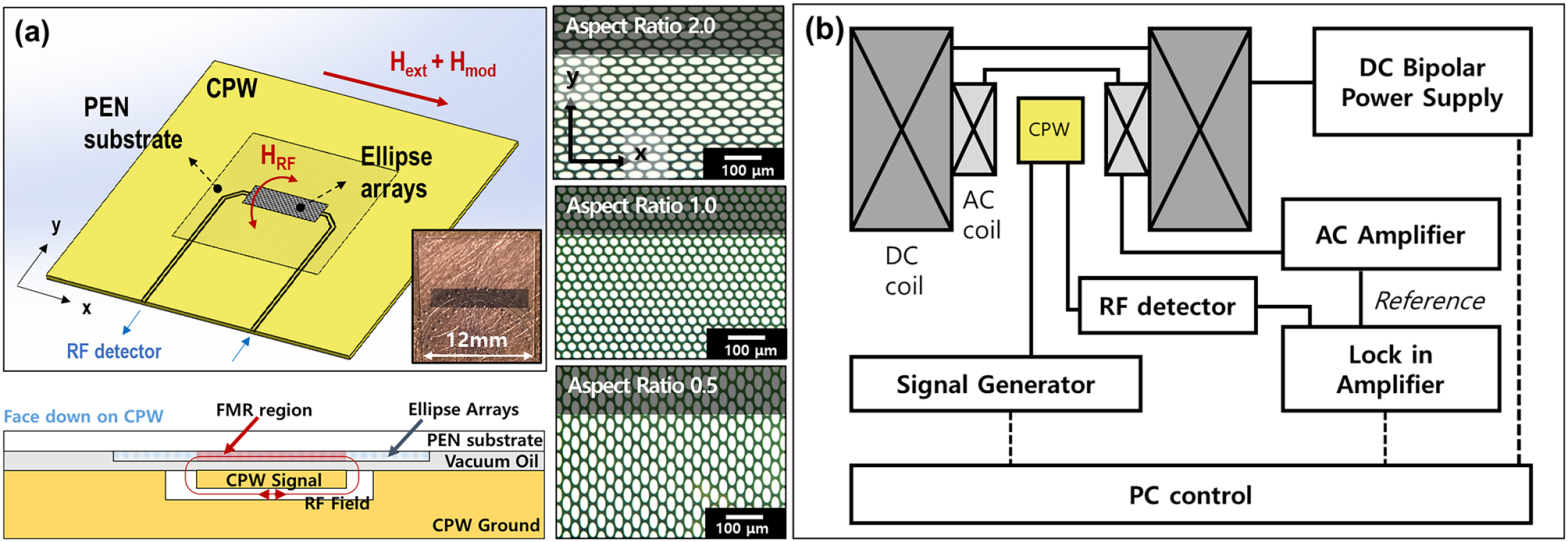
(**a**) Experimental setup for conducting ferromagnetic resonance (FMR) measurements using coplanar waveguide (CPW) on Permalloy ellipses with aspect ratios(width/length) of 2.0(40 μm/20 μm), 1.0(20 μm/20 μm), and 0.5(20 μm/40 μm) on a flexible substrate. (**b**) Schematic representation of the systematic approach for generating radio frequency signals in the coplanar waveguide and providing an intermediate frequency magnetic field and to the lock-in amplifier.

Figure 1a presents the meticulously crafted ellipse arrays featuring aspect ratios (width along the x-axis to height along the y-axis) of 2.0, 1.0, and 0.5. This diverse range of aspect ratios was chosen to systematically study the effects of shape anisotropy on the FMR properties of the arrays. To accurately evaluate their capability to absorb RF microwaves, the substrate was strategically placed upside down. This orientation brought the magnetic patterns into closer proximity with the signal channel of a coplanar waveguide (CPW), optimizing the interaction between the magnetization and the microwave field. In this configuration, the working frequency range was capped at 18 GHz, constrained by the RF cables and connectors. This range is considered adequate for performing FMR analyses on Permalloy thin films and patterns, accommodating a realistic magnetic field span (below 500 mT).

FMR was meticulously initiated by fine tuning the radio frequency and adjusting the dc magnetic field using electromagnets. To capture the microwave absorbing signals with high precision, a lower frequency of the ac magnetic field from a modulation coil was utilized, employing a lock-in technique to enhance the sensitivity and specificity of the detection. This specialized measurement setup for FMR detection using CPW is depicted in Fig. 1b.

**Figure 2:**
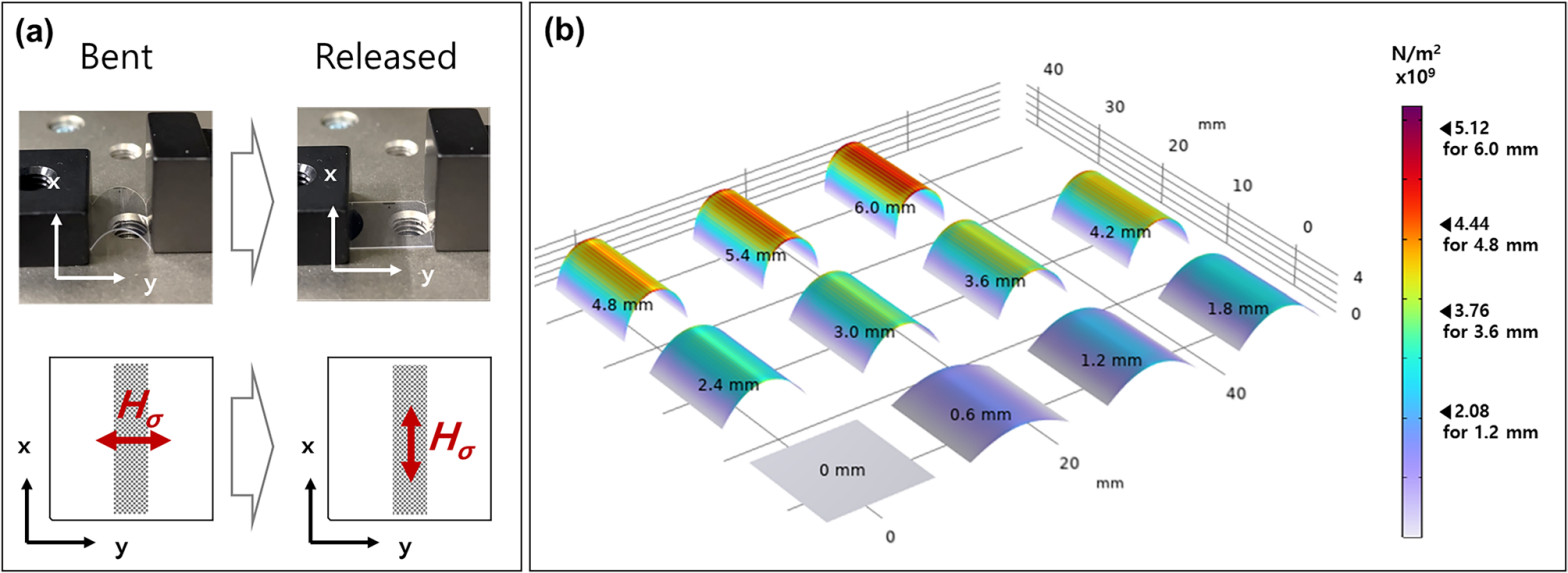
(**a**) Depiction of the bending geometry during both bent and released conditions using a motor controlled stage. (**b**) Simulation results illustrating the tensile stress on Permalloy thin film on the flexible substrate relative to the bending distance. The maximum tensile stress under bent condition are listed next to the color bar for bending distance of 1.2, 3.6, 4.8, and 6.0 mm.

The ellipse arrays were subjected to bending in the y-direction, introducing tensile stress to the patterns during the bending process. Previous investigations^34,41^ have convincingly shown that a reverse magnetostriction effect is observed upon the release of the stress. As a result, the ellipse arrays developed stress-induced magnetic anisotropy fields in the x-direction, attributable to the positive magnetostriction coefficient of Permalloy. This phenomenon allows for the deliberate manipulation of magnetic anisotropy through mechanical means. The precision in controlling the travel distance and the number of bending repetitions was ensured by employing a linear motorized stage, as depicted in Fig. 2a. This setup facilitated the application of consistent and repeatable stress across the samples.

During each bending cycle, the ellipses were subjected to alternating tensile and compressive stresses. Due to the positive magnetostriction coefficient inherent to Permalloy, the ellipses developed stress induced magnetic anisotropy fields oriented along the y-axis while in the bent state. In contrast, upon release from the bent condition, the orientation of the stress induced magnetic anisotropy field shifted to align along the x-axis. This dynamic response to mechanical stress highlights the tunable nature of the magnetic properties under investigation.

The most significant stress was localized in the central region of the PEN substrate, along the axis of bending, as illustrated in Fig. 2b. This distribution of stress provided the rationale for positioning the ellipse arrays centrally on the PEN substrate, ensuring that they were subjected to the maximal stress impact during the bending cycles. Each bending event was characterized by a fixed travel distance of 3.5 mm, translating to an approximate stress of $3.6 \times {10}^{9}$ N/m^2^ applied to the central region. In this 3D bending simulation, the key parameters for calculating stress were the Young’s Modulus and Poisson’s ratio of the PEN and metallic thin film. We used a Young’s Modulus of 2 GPa and a Poisson ratio of 0.4 for the PEN, and a Young’s Modulus of 200 GPa and a Poisson ratio of 0.29 for the metallic film, based on the materials library of 3D simulation. Because of the stress profile, we positioned the pattern arrays in the central region, where majority of the stress is applied and detected through FMR detection.

## Results and discussion

Before delving into the intricacies of stress induced magnetic anisotropy in the ellipse arrays, we embarked on a foundational characterization of a 27 nm thick Permalloy thin film, which was deposited on a SiOx substrate complemented with 3.6 nm tantalum layers both below and above the Permalloy. This initial step was critical for establishing a baseline understanding of the magnetic properties inherent to the materials employed in our study. The characterization was meticulously carried out using FMR detection techniques, which played a pivotal role in enabling us to execute more precise calculations concerning the magnetic anisotropies attributed to both the shape and stress within the patterned arrays.

**Figure 3:**
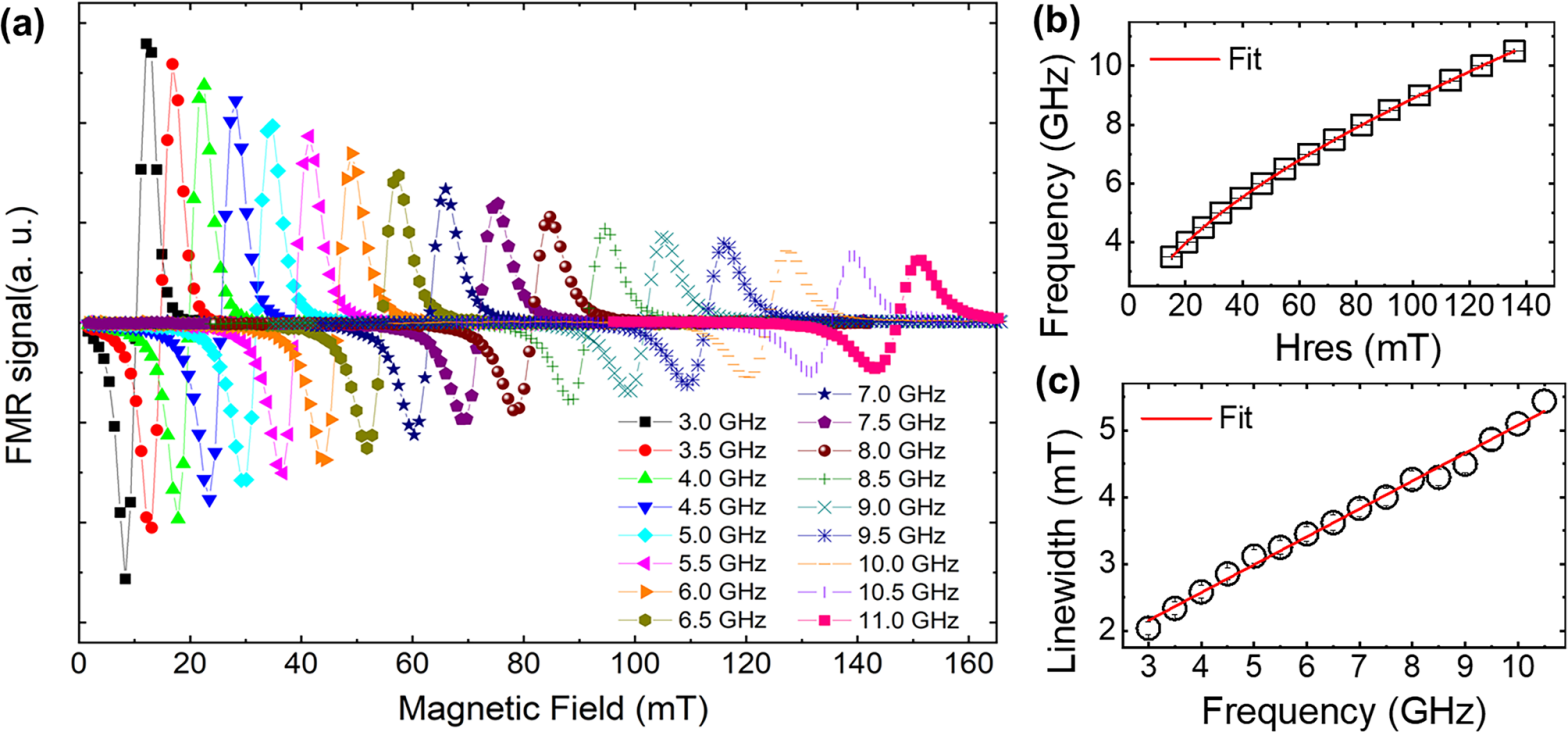
(**a**) shows Ferromagnetic Resonance (FMR) signals for Permalloy thin films, highlighting the resonance frequency range from 3.0 to 11.0 GHz. (**b**) This part depicts the relationship between resonance frequency and the resonance field in FMR detection, with the fit curve demonstrating Kittel’s equation. (**c**) This illustration presents the variation in the linewidth of FMR detection as it relates to the resonance frequency.

Figure 3a shows the FMR detection signals obtained from the non-patterned Permalloy film, with the microwave frequency in this particular set of measurements spanning from 3 to 11 GHz. The FMR signal at the resonance condition is proportional to the input microwave power. The RF detector voltage in the system follows a Lorentzian curves as a function of the magnetic field at a resonance frequency. Then, the modulation of the detection signal, brought about by an alternating magnetic field emanating from an ac coil, allowed the lock-in amplifier to generate field derivative curves corresponding to each applied external magnetic field. Therefore, the parameters for FMR peaks were obtained by symmetric (S) and antisymmetric (A) Lorentzian functions^42^,

2 $${\text{S}}\left( {\text{H}} \right) = {\raise0.7ex\hbox{${\delta H^{2} }$} \!\mathord{\left/ {\vphantom {{\delta H^{2} } {\left( {H - H_{res} } \right)^{2} + \delta H^{2} }}}\right.\kern-0pt} \!\lower0.7ex\hbox{${\left( {H - H_{res} } \right)^{2} + \delta H^{2} }$}},\;A\left( {\text{H}} \right) = {\raise0.7ex\hbox{${\left( {H - H_{res} } \right)\delta H}$} \!\mathord{\left/ {\vphantom {{\left( {H - H_{res} } \right)\delta H} {\left( {H - H_{res} } \right)^{2} + \delta H^{2} }}}\right.\kern-0pt} \!\lower0.7ex\hbox{${\left( {H - H_{res} } \right)^{2} + \delta H^{2} }$}}\user2{ }$$

The Fits involves the key parameter of resonance field $\left( {H_{res} } \right)$, the half-linewidth $\left( {\delta H} \right)$. With the definitions from voltages in the lock-in amplifier, the outputs were given by

3 $${{V}}_{{{X}}} \left( {{H}} \right) = \user2{ }{{{\Gamma}}}\left[ { {{S}}\left( {{H}} \right){{cos}}(\emptyset_{{{S}}} } \right) + {{A}}\left( {{H}} \right){{sin}}(\emptyset_{{{S}}} )]$$

4 $${{V}}_{{{Y}}} \left( {{H}} \right) = \user2{ }{{{\Gamma}}}\left[ {{{S}}\left( {{H}} \right){sin} (\emptyset_{{{S}}} ) - {{A}}\left( {{H}} \right){cos}(\emptyset_{{{S}}} )} \right]$$

This approach provided a clear way to observe the magnetic behavior of Permalloy thin film. Figure 3b and c present, respectively, the resonance field and the linewidth ascertained under FMR conditions. Through the application of Kittel’s equation and leveraging established theoretical model, $\delta {\text{H}} = \delta H_{0} + \alpha \left( {2\pi f/\gamma_{e} } \right)$, for damping constant, we were able to derive fitted curves from the data. Here, ${\delta H}$ is half-linewidth of the FMR signal, and $\alpha$ is damping constant. These curves revealed a saturation magnetization (M_S_) of 703.5 emu/cm^3^ and a damping constant (α) of 0.0117. Notably, these parameters align closely with findings previously reported in the literature^18,41^, thereby validating our experimental methodology and the robustness of our analysis.

**Figure 4:**
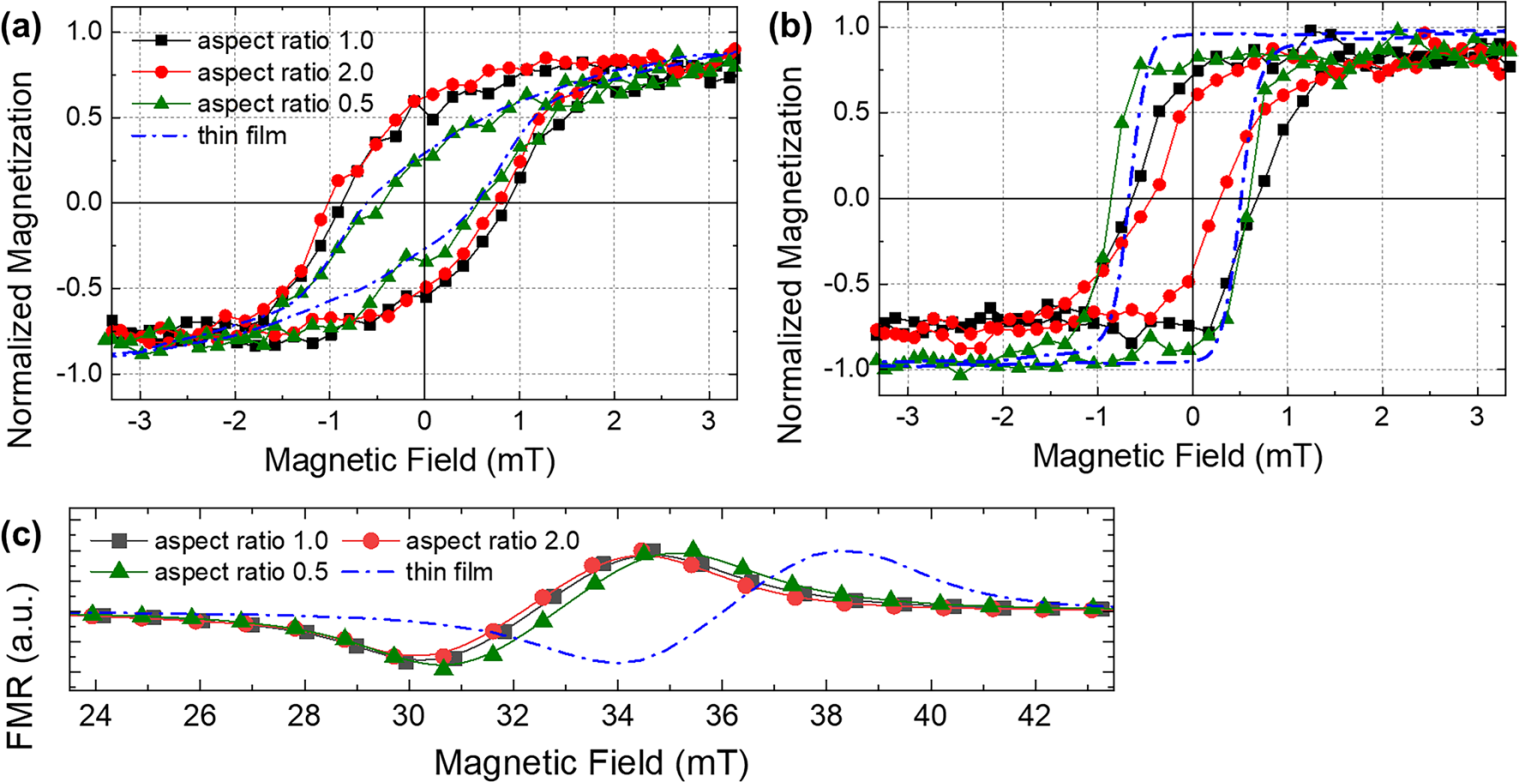
(**a**) and (**b**) show normalized magnetization curves through VSM (Vibrating Sample Magnetometer) detection for the thin film and the elliptical arrays subjected to an external magnetic field applied along the (**a**) x-direction (hard axis) and (**b**) y-direction (easy axis), respectively. (**c**) illustrates variation of the ferromagnetic resonance field at 5 GHz as a function of the aspect ratio of the ellipses, and continuous thin film.

The normalized magnetization curves of the ellipse arrays through VSM (Vibrating Sample Magnetometer, Lakeshore 7400 model) detection concerning the external magnetic field are depicted in Fig. 4a and b, with the magnetic field applied along the x- and y-axes, respectively. Because we applied the magnetic field of 20 mT (deposition field) along the y-axis during the sputtering, the hysteresis loops in the Fig. 4b exhibit a more squared curves compared to those in the Fig. 4a. Applying an external magnetic field during the sputtering process influences the direction of the magnetic moment alignment within the film, creating a preferred axis of the magnetization. Therefore this process can produce induced uniaxial anisotropy field, where the easy-axis of magnetization is aligned along the direction of the applied field. This technique effectively tailors the magnetic properties of the film, particularly the directional dependence of its magnetization, to enhance its application potential in various magnetic devices. To illustrate the differences in magnetization reversal behaviors of the Permalloy thin film, blue dot-dashed lines have been added to Fig. 4a and b. These clearly demonstrate how the magnetization behaviors were altered by the additional magnetic anisotropy introduced by the pattern’s shape. The squareness (remanence/saturation magnetization) of the ellipse exhibited values of 0.25, 0.5, and 0.6 under an external magnetic field in the x-direction, corresponding to aspect ratios of 0.5, 1.0, and 2.0, respectively. When the external magnetic field was applied in the y-direction, the squareness decreased from 0.8 to 0.55 as the aspect ratio of the ellipse increased from 0.5 to 2.0. The saturation magnetization of Permalloy thin film, measured by VSM at 695.2 emu/cm^3^ either in x- or y- direction, closely aligns with the FMR derived result of 703.5 emu/cm^3^.

Subsequently, we investigated the microwave absorption properties depending on the shape anisotropy from the aspect ratio of the ellipse. Based on the demagnetization field theory, our ellipses were considered as the oblate spheroid, because of very thin thickness of the Permalloy comparing to width and length of the ellipses. Therefore, the demagnetizing factors could be defined as:

5 $$N_{z} = \frac{{4\pi r^{2} }}{{r^{2} - 1}}\left( {1 - \sqrt {\frac{1}{{r^{2} - 1}}} \mathbf{sin}^{ - 1} \left( {\frac{{\sqrt {r^{2} - 1} }}{r}} \right)} \right),\;N_{x} \approx { }N_{y} = \frac{{\pi^{2} }}{r}$$

$r$ is width (or length) / thickness of the ellipse. When N_x= N_y, the sole remaining anisotropy term will be due to the induced uniaxial anisotropy. In our ellipse geometry, $r$ could be estimated as 740.74 for 20 μm $\left( {N_{long} } \right)$ and 370.37 for 10 μm $\left( {N_{short} } \right)$, respectively. In the thin film geometry, considering that the primary demagnetization field $\left( {\sim { }4{\uppi }M_{S} } \right)$ aligns along the z-axis (perpendicular to the film plane), the magnitude of the in-plane shape anisotropy field was relatively minor, yet detectable.

Employing the uniaxial magnetic anisotropy model^18^, we simplified the calculation of the overall magnetic anisotropy field $\left( {H_{eff} } \right)$ taking into account both the aspect ratio and induced anisotropy from deposition field via total energy calculation (*E*):

6 $$E = - M_{S} H\ \mathbf{sin} \left( \theta \right) + K_{U} \mathbf{sin}^{2} \left( \theta \right) + \frac{1}{2}M_{S}^{2} \left( {N_{x} \mathbf{sin}^{2} \left( \theta \right) + N_{y} \mathbf{cos}^{2} \left( \theta \right) + N_{z} } \right)$$

where $K_{U}$ is induced anisotropy energy from the deposition, θ is the angle between the saturationmagnetization $\left( {M_{S} } \right)$ and the deposition field direction (y-axis) in the film plane. $N_{i}$ is the demagnetization factor from Eq. (5) in the x- or y-direction. From the equilibrium condition $\left( {\partial {\text{E }}/\partial {\uptheta } = 0} \right)$, the effective field, which includes induced anisotropy and shape anisotropy, could be defined as:

7 $$H_{eff} = \left( {\frac{{2 K_{U} }}{{M_{S} }} + M_{S} \left( {N_{x} - N_{y} } \right)} \right)\mathbf{sin}\left( \theta \right)$$

The effective magnetic field, a sum of shape and induced anisotropy fields, determines the overall magnetic behavior of the ellipse arrays under external magnetic fields. For an ellipse with an aspect ratio of 0.5 under the external magnetic field in the x-axis $\left( {\theta = \pi /2} \right)$, the effective magnetic field is now defined by the Eq. (7) and observed as approximately 2.1 mT in the Fig. 4a.

During FMR detection, we also observed consistent alteration in the resonance field that corresponded with changes in the effective magnetic field as the aspect ratio of the ellipse array was varied from 0.5 to 2.0. The observed alteration in the resonance field, which was maintained across a constant resonance frequency, was induced by the application of an external magnetic field along the x-axis. Figure 4c illustrates the normalized FMR signals of the Permalloy ellipse arrays in relation to the aspect ratio and continuous thin film, with the resonance frequency set at 5 GHz. Comparing to the VSM measurements, the continuous thin film and the array with an aspect ratio of 0.5 showed similar magnetic switching behaviors under external magnetic field in both the x- and y-directions. However, the resonance field shifted toward smaller values, likely due to anisotropy variations resulting from the patterned shapes. The effective magnetic field $\left( {H_{eff} } \right)$ of 2.0 mT is quantified using the Kittel’s model, $f_{res}^{2} /\left( {\gamma_{e}^{2} \left( {H_{0} + 4{\uppi }M_{S} } \right)} \right) - H_{0}$, incorporating the measured saturation magnetization (703.5 emu/cm^3^) and the resonance field of 32.8 mT for the ellipse array with an aspect ratio of 1.0. This calculated effective field aligns well with the value derived from the hysteresis loops shown in Fig. 4a. Due to the consistent induced anisotropy applied to the ellipses in the y-direction, the difference in the effective magnetic field between the ellipse arrays with aspect ratio of 0.5 and 2.0 is attributed to the difference in the shape magnetic anisotropy field, expressed as $2\pi^{2} M_{S} \left( {\frac{1}{{N_{long} }} - \frac{1}{{N_{short} }}} \right)$ and calculated as 1.927 mT. Therefore, we estimate that the increase in the effective magnetic field, depending on the aspect ratio (2.0, 1.0, 0.5) of the ellipse array, is approximately 0.8 mT and shown in Fig. 4c. As we discussed in Eq. (5), the demagnetizing factor in either x- or y- is two order smaller than the demagnetizing factor in z-direction. Therefore, the effective magnetic field is quantified primarily by the induced anisotropy field, which is approximately 20 mT, along with a smaller contribution from the shape anisotropy field. The consistent changes in the resonance field, corresponding to variations in the effective magnetic field, demonstrate the precise control achievable over the film’s magnetic behavior through the application of an external magnetic field during sputtering and the shape of the patterns. This control is crucial for the design and optimizing ferromagnetic thin films for advanced applications in magnetic sensing, storage, and other spintronic devices. The findings not only validate the net anisotropy effects but also open up avenues for further exploration into the tailored magnetic properties of ferromagnetic thin films through deposition field and shape.

**Figure 5:**
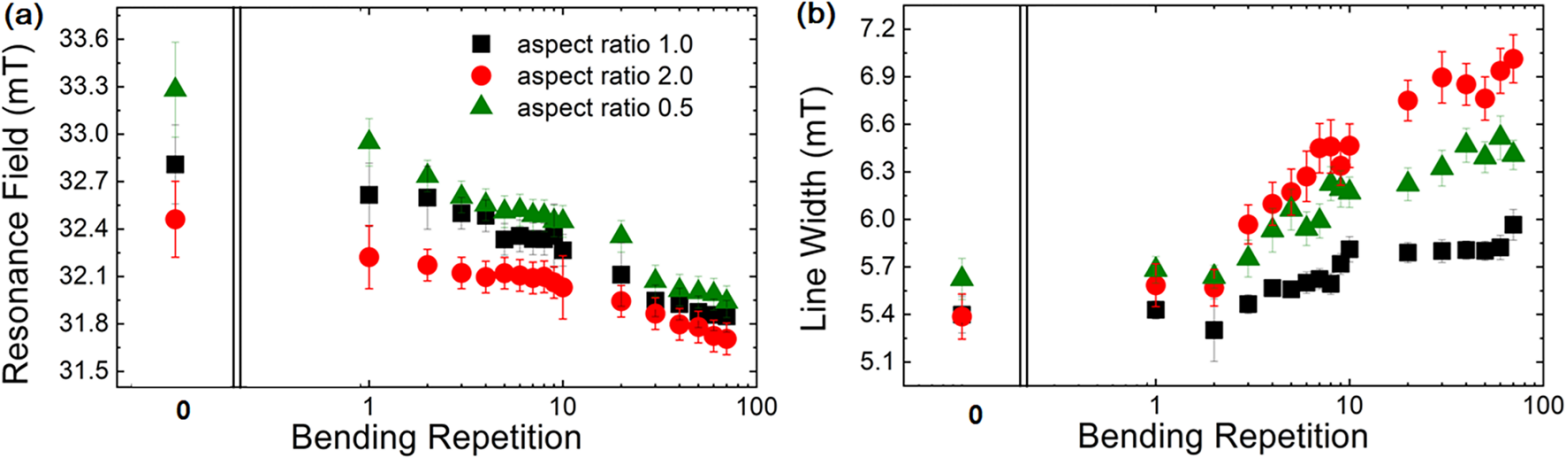
The impact of the aspect ratio on how the resonance field changes (**a**) and the linewidth of ferromagnetic resonance signal (**b**) with each increase in bending repetitions for elliptical structures is contingent on the aspect ratio.

As previously mentioned, the application of external stress on magnetic patterns or thin films generates an additional anisotropy field through the magneto-mechanical effect. Owing to positive magnetostriction coefficient of Permalloy, a stress induced anisotropy field is created along the x-axis when the flexible patterns is released from a bent condition. As the number of bending repetitions increased, the accumulation of stress induced magnetic anisotropy fields progressed, ultimately reaching saturation. This led to a shift in the resonance fields of all samples towards lower values at a constant RF of 5 GHz. The resonance field shifts from the bending repetition depending on the aspect ratio of ellipse arrays are represented in the Fig. 5. The first bending of all samples induced a big shift from the initial resonance field toward smaller field. This could be thought that the strong mechanical bending induced a strong inverse-magnetostriction effect on the patterns so that the magnetic domains received additional anisotropy field toward x-direction after bending^41^. Therefore, this big shift brings the notable variation in the resonance field or resonance frequency in the patterns to modify the measuring magnetic field or absorbing the microwave frequency. With increasing the bending repetition up to 10 cycles, the resonance field shift varied gradually. In this region, the manipulation of the resonance field or absorbing frequency is possible with increasing the bending repetition. With further increase of bending repetition, the variation of the resonance field getting smaller and saturated. It would be very useful for the applications that are not sensitive to external bending stress and bending repetitions to measure the resonance field or absorbing microwave frequency. The shift in resonance field, plotted on a logarithmic scale for up to 70 bending cycles, is represented in Fig. 5a. This phenomenon highlights the ability to adjust the microwave absorption or sensing frequency by systematically introducing stress and shape induced magnetic anisotropy fields through repeated bending. With each increase in bending repetitions, the FMR signal linewidth experienced broadening, as shown in Fig. 5b. Initially, it is believed that the broadening stems from cracks forming along the y-direction as a result of bending. These micro-morphological alterations, through the introduction of cracks, could potentially modify boundaries, which is expected to inversely affect the damping mechanisms, contributing to the broadening of the FMR signal linewidth observed. Consequently, this phenomenon is predominantly attributed to the inverse-magnetostriction effect (or MME), enhancing the material’s damping response to external magnetic fields.

## Conclusions

The results highlight the ability to precisely adjust the FMR frequency or magnetic field within ellipse arrays by leveraging magnetic anisotropy fields that originate from both the geometrical configuration of the patterns and the mechanical stress introduced by bending. The role of aspect ratio in influencing magnetic anisotropy within these arrays was validated through two key observations: changes in magnetization reversal and alterations in microwave absorption frequency. When the magnetic field was applied in the x-direction of the ellipse arrays, the squareness varied as follows: 0.25 for an aspect ratio of 0.5, 0.5 for an aspect ratio of 1.0, and 0.6 for an aspect ratio of 2.0. Conversely, the ferromagnetic resonance frequency decreased from 33.28 mT to 32.56 mT as the aspect ratio of the ellipse increased from 0.5 to 2.0. The presence of stress induced magnetic anisotropy was confirmed by observing shifts in the ferromagnetic resonance field after each release, with up to 70 bending repetition. All ellipse arrays, with aspect ratio of 0.5, 1.0 and 2.0, showed approximately 6% decrease in the resonance field after 70 bending repetitions. The study achieved that a precisely shift of the resonance field under FMR condition by applying the physical shape of ellipse arrays and the number of bending repetition. The initial condition for microwave absorption was set by the shape anisotropy resulting from the aspect ratio of the ellipses, whereas the stress induced magnetic anisotropy altered the effective magnetic field within the ellipses.

In summary, the outcomes of this study reveal the ability to finely tune the absorbing microwaves selectively, influenced by the pattern’s geometry and repeated bending, to modify the absorption frequency or magnetic field for shifts to either lower or higher frequency ranges. This adaptability opens exciting prospects for enhancing RF based spintronic devices, including sensors, absorbers, and antennas, by providing a novel method for their precise control and optimization.

## Data Availability

All data generated or analyzed during this study are included in this published article.
